# Multimolecular Competition Effect as a Modulator of Protein Localization and Biochemical Networks in Cell‐Size Space

**DOI:** 10.1002/advs.202308030

**Published:** 2023-12-06

**Authors:** Saki Nishikawa, Gaku Sato, Sakura Takada, Shunshi Kohyama, Gen Honda, Miho Yanagisawa, Yutaka Hori, Nobuhide Doi, Natsuhiko Yoshinaga, Kei Fujiwara

**Affiliations:** ^1^ Department of Biosciences and Informatics Faculty of Science and Technology Keio University 3‐14‐1 Hiyoshi, Kohoku‐ku Yokohama Kanagawa 223‐8522 Japan; ^2^ Komaba Institute for Science Graduate School of Arts and Sciences The University of Tokyo Komaba 3‐8‐1 Meguro Tokyo 153‐8902 Japan; ^3^ Graduate School of Science The University of Tokyo Hongo 7‐3‐1 Bunkyo Tokyo 113‐0033 Japan; ^4^ Center for Complex Systems Biology Universal Biology Institute The University of Tokyo Komaba 3‐8‐1 Meguro Tokyo 153‐8902 Japan; ^5^ Department of Applied Physics and Physico‐informatics Faculty of Science and Technology Keio University 3‐14‐1 Hiyoshi, Kohoku‐ku Yokohama Kanagawa 223‐8522 Japan; ^6^ WPI Advanced Institute for Materials Research (WPI‐AIMR) Tohoku University Katahira 2‐1‐1, Aoba‐Ku Sendai 980‐8577 Japan; ^7^ MathAM‐OIL AIST Sendai 980‐8577 Japan; ^8^ Present address: Department for Cellular and Molecular Biophysics Max Planck Institute for Biochemistry Am Klopferspitz 18 D‐82152 Martinsried Germany

**Keywords:** artificial cells, bottom‐up synthetic biology, cell‐size space effect, in vitro reconstitution, multimolecular effect

## Abstract

Cells are small, closed spaces filled with various types of macromolecules. Although it is shown that the characteristics of biochemical reactions in vitro are quite different from those in living cells, the role of the co‐existence of various macromolecules in cell‐size space remains still elusive. Here, using a constructive approach, it is demonstrated that the co‐existence of various macromolecules themselves has the ability to tune protein localization for spatiotemporal regulation and a biochemical reaction system in a cell‐size space. Both experimental and theoretical analyses reveal that enhancement of interfacial effects by a large surface‐area‐to‐volume ratio facilitates membrane localization of molecules in the cell‐size space, and the interfacial effects are alleviated by competitive binding to lipid membranes among multiple proteins even if their membrane affinities are weak. These results indicate that competition for membrane binding among various macromolecules in the cell‐size space plays a role in regulating the spatiotemporal molecular organization and biochemical reaction networks. These findings shed light on the importance of surrounding molecules for biochemical reactions using purified elements in small spaces.

## Graphical abstract

In confined cell‐size spaces, membrane localization of proteins is enhanced due to the cell‐size space effect. This enhanced membrane localization disturbs biochemical networks such as spatiotemporal regulation and protein expression. It is found that the competitive binding of surrounding macromolecules to membranes cancels this effect, ensuring the functionality of biochemical systems.

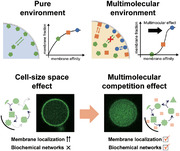

## 1 Introduction

Living cells are micro‐size spaces filled with various biomolecules such as genomic DNA, mRNA, and proteins (≈4000 species in *Escherichia coli*). Because of the diversity of biomolecules, purification, and characterization of individual biomolecules are crucial steps in analyzing biochemical systems in living cells. With the success of these reductionist strategies, the molecular mechanisms of various biochemical systems have been unveiled.^[^ ^1^ ^]^ However, it has been known that in vitro purified biomolecules do not necessarily show the same behaviors as those under the in vivo environment.

Although macromolecular crowding has been identified as the cause of this difference between in vitro and in vivo,^[^ ^2^ ^]^ another important feature of living cells is the confinement of biomolecules in a micrometer‐sized space. In living cells, thousands of biomolecules are confined by lipid membranes in a space with a diameter of 1–100 µm.^[^ ^3^ ^]^ Remarkable features of these small spaces (called cell‐size spaces) include a small number of elements inside the space and a large surface‐area‐to‐volume ratio.^[^ ^4^ ^]^ Owing to the small volume in the cell size space, the number of molecules is limited and fluctuates among cells, which can lead to drastic state transitions in molecular assembly and cell‐to‐cell variability.^[^ ^5^ ^]^ A large surface‐area‐to‐volume ratio makes the interfacial effects more apparent, promoting the chemical effects of lipid membranes.^[^ ^6^ ^]^ The elucidation of these effects (referred to as the cell‐size space effect)^[^ ^4b^ ^]^ is necessary to understand the fundamental differences in the behavior of biomolecules in vitro and in cells.

To elucidate the cell‐size space effect, biochemical systems reconstituted with purified biomolecules have been entrapped within a cell‐size space covered with lipid membranes. These studies have revealed how cell‐size space affects them.^[^ ^1^, ^3^, ^6^, ^7^ ^]^ For instance, these studies have shown that cell‐size space effects confer specific characteristics to molecular diffusion dynamics,^[^ ^8^ ^]^ DNA or protein localization,^[^ ^3a^ ^]^ gene expression,^[^ ^6^, ^9^ ^]^ and higher‐order structures of cytoskeleton proteins such as actomyosin.^[^ ^7^, ^10^ ^]^ These studies revealed how the characteristics of biochemical systems in cell‐size spaces differ from those of test tube reactions.

Despite the success of the investigation of cell‐size space effects, several studies have suggested that some characteristics of biochemical systems in the cell‐size space do not match those in living cells.^[^ ^6^, ^9^ ^]^ For example, our recent reconstitution studies have shown that a bacterial cell division plane determination system (Min system) and transcription‐translation do not work correctly in cell‐size spaces covered with physiological lipids.^[^ ^6^, ^9^ ^]^

Min system determines the cell division plane using a dynamic wave via reaction‐diffusion coupling of two proteins (MinD and MinE) in *E. coli* cells. MinD attaches to the membrane by binding to ATP in the cytosol and recruits another MinD to the lipid membranes. MinE interacts with membrane‐bound MinD and stimulates the ATPase activity of MinD. MinD dissociates from membranes, and binds to ATP in the cytosol again. This reaction cycle is coupled with differences in the molecular diffusion rates in the cytosol and membrane, resulting in a spatiotemporal protein gradient on the membrane called Min waves.^[^ ^11^ ^]^ In our previous report, we found that MinE spontaneously localizes on lipid membranes without MinD via the cell‐size space effect, and suppression of this spontaneous MinE membrane localization is essential to generate Min waves in cell‐size spaces covered with *E. coli* polar lipids.^[^ ^6b^ ^]^ PURE system, a reconstituted transcription‐translation system that uses dozens of purified elements,^[^ ^12^ ^]^ is also inactivated by the cell‐size space effect. Our previous study showed that protein expression levels of PURE system in a cell‐size space covered with *E. coli* polar lipids were significantly lowered than those in the tube reaction.^[^ ^9b^ ^]^ In both cases, we found that supplementation with BSA restored the function of Min and PURE systems in the cell‐size space.^[^ ^6^, ^9^ ^]^ However, BSA is not present in bacterial cells and does not appear to be related to the Min and PURE systems. Furthermore, the biochemical mechanisms and elements are different between the two systems. Hence, this coincidence among different biological systems suggests that surrounding proteins in the cell‐size space play a universal role in ensuring their correct function in living cells.

Here we show how surrounding proteins affect biochemical systems in the cell‐size space by using reconstituted systems. By the assays using biochemical fractionation of cell extracts and purified proteins, we found that many proteins have similar effects to BSA in both Min and PURE systems. Moreover, both experimental and theoretical analyses revealed that the levels of the effect were related to the enhanced interfacial effects due to the high surface‐area‐to‐volume ratio and resource limitation of membrane binding sites in the cell‐size space. Consequently, we concluded that competition for membrane sites by weakly binding proteins can alter the reaction behavior of biochemical systems in the cell‐size space. These results show that competitive membrane binding occurring in multimolecular environments (multimolecular competition) plays an essential role in regulating biological systems, and suggests that its consideration is necessary to depict faithful traits of biological systems in the cell‐size space.

## 2 Results

### 2.1 Various *E. coli* Proteins Show BSA‐Like Effect on Min Wave Generation in Cell‐Size Space

Although supplementation with BSA drastically changes the dynamics of Min and PURE systems,^[^ ^6^, ^9^ ^]^ the actual role of BSA remains still elusive. The cell‐size space effect inhibits Min wave generation because of the spontaneous lipid membrane localization (Slim) of MinE due to the cell‐size space effect derived from the high membrane‐area‐to‐volume ratio.^[^ ^6b^ ^]^ Due to Slim of MinE (Slim‐MinE), MinE highly accumulates on membranes in the cell‐size space. Slim‐MinE disrupts the reaction balance between MinD and MinE to generate Min waves. Therefore, the suppression of Slim‐MinE is essential for Min wave emergence in the cell‐size space and BSA is its suppressor.^[^ ^6^, ^13^ ^]^ However, the reason why BSA suppresses Slim‐MinE was not determined. As a clue to address this question, we previously found that cell extract of *E. coli* showed a similar effect as BSA on Min wave generation in the cell‐size space.^[^ ^6b^ ^]^ Therefore, we first focused on the effect of BSA on Min waves reconstituted in the cell‐size space (Movie S1, Supporting Information).

Water‐in‐oil microdroplets covered with lipids were used as an emulator of the cell‐size space (Figure S1, Supporting Information). Although this microdroplet system is based on monolayer lipid membranes, it has been used as an emulator to elucidate cell‐size space effects.^[^ ^4^, ^6^, ^7^, ^14^ ^]^ *E. coli* polar lipids were used as the interface of the microdroplets throughout this study unless otherwise specified. This microdroplet system was the same as that used in our previous Min wave studies.^[^ ^6^, ^13^ ^]^ Unless otherwise specified, microdroplets with a diameter of 10–30 µm were randomly selected and analyzed (see Experimental Section). To clarify the cause of Slim‐MinE suppression by the microdroplet system, we biochemically fractionated *E. coli* cell extracts to search for proteins that exhibit a BSA‐like role in Min wave generation in the cell‐size space.

**Figure 1:**
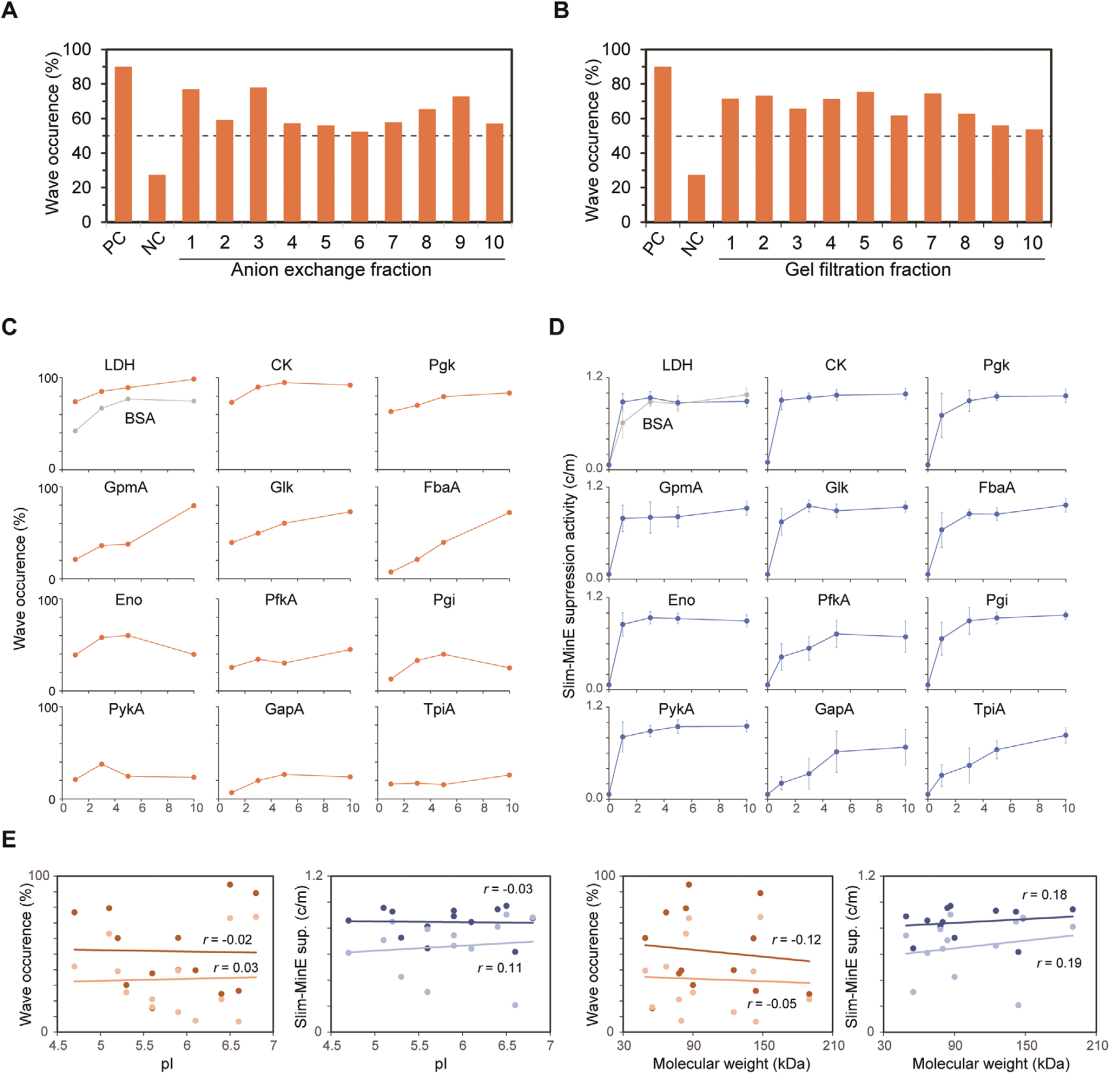
Various proteins have the ability to generate Min wave in cell‐size space. A,B) Min wave occurrences (percentages of artificial cells with Min waves) by supplementation of cell extract fractions instead of BSA (*n* = 100–125). PC: 3 mg mL^−1^ *E. coli* cell extract, NC: RE buffer is added instead of each fraction (See Experimental Section). 0.4 mg mL^−1^ CK was added in all samples including positive and negative controls. A) Fractions of ion exchange (0.2 mg mL^−1^ at final) were added to the Min reaction mixture instead of BSA. B) Fractions of gel filtration (0.2 mg mL^−1^ at final) were added to the Min reaction mixture instead of BSA. Dotted lines indicate 50% of artificial cells with Min waves. C) Min wave occurrences and D) Slim‐MinE suppression determined by cytosol/membrane intensity ratio (c/m) of MinE in cell‐size microdroplets (10–30 µm) when various concentrations of 12 purified proteins were supplemented instead of BSA. Gray line indicates Slim‐MinE suppression activity in the case of BSA supplementation. C) Min wave occurrences (*n* = 97–128) in the case of encapsulating 1 µm His‐msfGFP‐MinD, 1 µm MinE‐mCherry‐His, and each purified protein. D) Slim‐MinE suppression activity (c/m) was calculated from the fluorescence intensity of microdroplets containing 1 µm MinE‐mCherry‐His and each purified protein (*n* = 10, average ± SD). E) Relationship between properties of the proteins (pI and molecular weight) and Min wave occurrence or Slim‐MinE suppression activity. Generation rates of Min waves by 1 (light colors) or 5 mg mL^−1^ (dark colors) additional proteins were plotted against the molecular weight (left) or isoelectric point (light) of purified proteins. Pearson correlation coefficients were shown as *r*.

*E. coli* cell extract was fractionated into 10 fractions by anion exchange or gel filtration which separated the proteins by electronic charges or molecular weight, respectively. After fractionation, 0.2 mg mL^−1^ (at final) of each fraction was mixed with Min proteins and then encapsulated in a cell‐size space. We should note that 0.4 mg mL^−1^ creatine kinase (CK) was supplemented in the Min reaction mixture to maintain ATP levels even for the negative control, and therefore, the total protein concentration added to the mixture was 0.6 mg mL^−1^. Surprisingly, Min waves were observed in all tested fractions (*p* < 10^−6^, binominal test) irrespective of anion exchange (**Figure** **1**A) or gel filtration (Figure 1B). These results indicate that the BSA‐like effect on Min wave is a general property of various proteins and that the effect is not derived from molecular sizes or total charges of proteins.

To test whether various proteins truly induce Min wave generation in the cell‐size space, we next tested the effect of purified proteins on Slim‐MinE and Min wave emergence. For this purpose, we purified proteins such as 10 glycolysis enzymes (Pgk, FbA, GpmA, Glk, PfkA, Eno, Pgi, PykA, GapA, TpiA) from *E. coli*, lactate dehydrogenase (LDH) from *Bacillus*, and CK from chicken as abundant cytoplasmic proteins of living cells, and analyzed their effect on Min waves generation (Figure 1C). Hereafter, 0.4 mg mL^−1^ CK was not added unless otherwise stated. When 1–10 mg mL^−1^ of each purified protein was added to the reaction mixture, we found 7 out of 12 purified proteins generated Min waves in 50% or more of the microdroplets (*p* < 10^−6^, binominal test), and the probability of Min wave generation increased with concentration. Next, the purified proteins were mixed with MinE, and their ability to suppress Slim‐MinE was analyzed. Similar to the Min wave generation, the purified proteins showed Slim‐MinE suppression in a concentration‐dependent manner (Figure 1D). Therefore, we conclude that the role of BSA in Min system is a general property of many proteins.

The levels of Min wave generation and Slim‐MinE suppression were not significantly correlated (*p* > 0.05, no‐correlation test, *n* = 12) with the molecular weight or isoelectric point of the added proteins, as expected from the results of the fractionation assay (Figure 1E). Among the tested proteins that did not have the ability to generate Min waves in cell‐size space (Eno, Pgi, PykA, PykA, GapA, TpiA), Slim‐MinE was suppressed when supplied at high concentrations. It should be noted that Slim‐MinE suppression is not the only factor for Min wave generation in cell‐size space and other factors, such as conformational equilibrium of MinE and MinDE concentration, are also associated with Min wave generation,^[^ ^13^ ^]^ although the reason for this has not yet been clarified.

Because MinE is known to be localized in the cytosol in the absence of MinD in living cells,^[^ ^11b^ ^]^ Slim‐MinE seems to be derived from a weak interaction, typically classified as a non‐specific interaction in the biological context, between MinE and lipid membranes, which is enhanced by the cell‐size space effect as suggested in our previous study.^[^ ^6b^ ^]^ However, it remains unclear whether the membrane localization of strong membrane‐binding proteins, such as MinD, is also suppressed by the addition of purified proteins. To investigate this, MinD was mixed with purified proteins and encapsulated in the cell‐size space. None of the tested proteins changed the membrane localization of MinD (Figure S2, Supporting Information), indicating that the suppression of Slim‐MinE by the purified proteins was due to the inhibition of weak interactions between MinE and the lipid membranes.

### 2.2 Nonspecific Interaction Between Lipid Membranes and Proteins is the Key Factor in Min Wave Generation and Slim‐MinE Suppression

**Figure 2:**
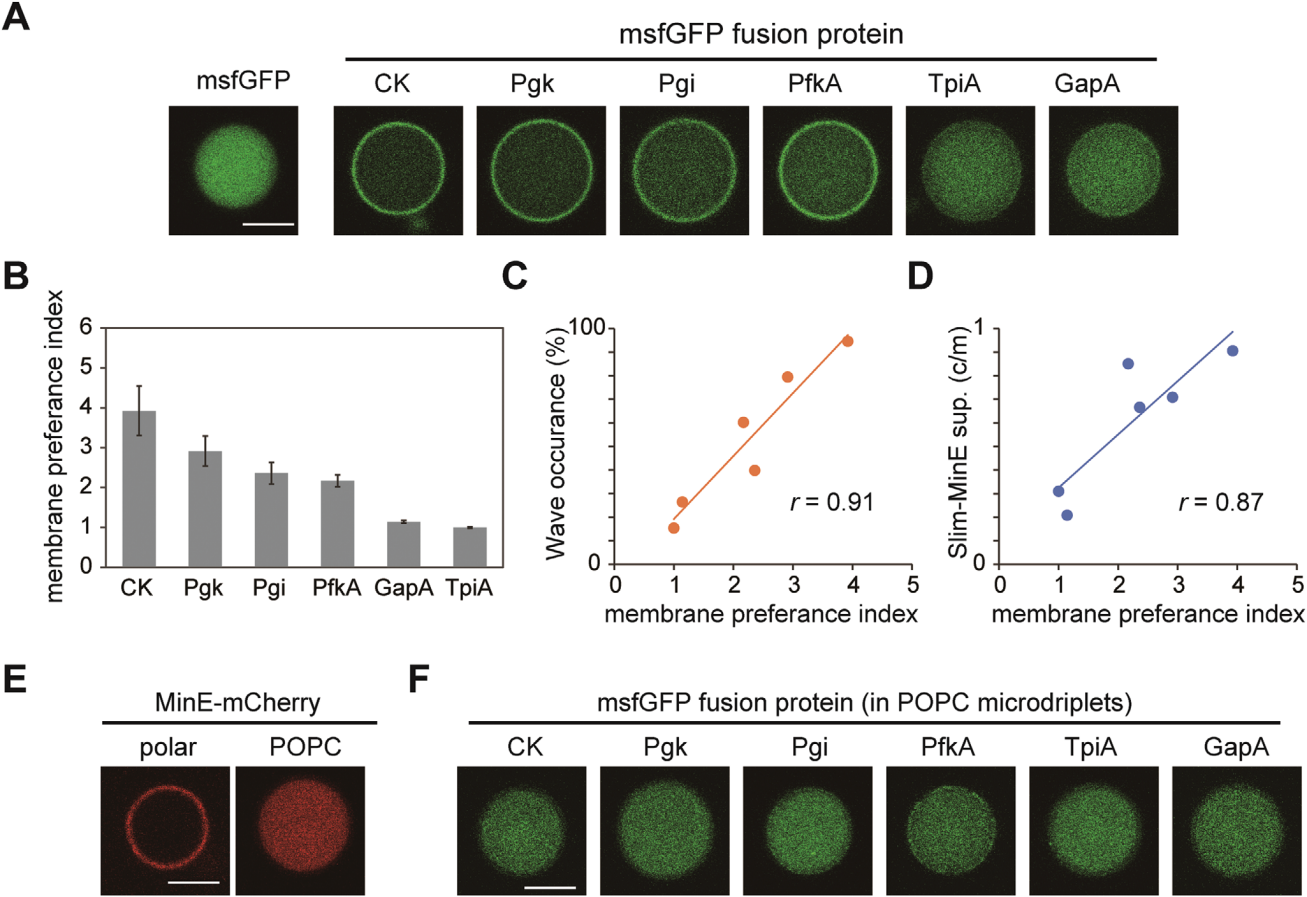
Various purified proteins spontaneously localize on membranes in cell‐sizes space. A) Confocal images of msfGFP‐fused purified proteins (0.2 mg mL^−1^) encapsulated in cell‐size microdroplets covered with *E. coli* polar lipids. Scale bar indicates 10 µm and all images are shown in the same pixels. B) Membrane preference index calculated from microdroplets with a diameter of 10–30 µm was shown (*n* = 8–10, average ± SE). C) Plots of percentages of artificial cells with Min waves by 5 mg mL^−1^ purified proteins against the membrane preference index of each protein. D) Plots of Slim‐MinE suppression activity (c/m) by 10 mg mL^−1^ purified proteins were supplied against the membrane preference index of each protein. Pearson correlation coefficients were shown as *r*. E) Confocal images of MinE‐mCherry‐His (0.2 mg mL^−1^) encapsulated in cell‐size microdroplets covered with *E. coli* polar lipids or a non‐charged lipid (POPC). F) Confocal images of msfGFP‐fused purified proteins encapsulated in cell‐size microdroplets covered with POPC. Scale bar indicates 10 µm and all images are shown in the same pixels.

A possible mechanism for the suppression of Slim‐MinE by the purified proteins is that the co‐supplemented proteins competitively bind to lipid membranes with MinE. In this case, purified proteins interact with the lipid membrane. To check this point, msfGFP was fused to the N‐terminal of proteins with various Slim‐MinE activity (CK, Pgk, Pgi, PfkA, GapA, and TpiA) and analyzed to determine whether the msfGFP fusion proteins show spontaneous lipid membrane binding by the cell‐size space effect. All tested proteins were found in the cytosol of living cells (Figure S3, Supporting Information) and seemed to have little affinity for the lipid membrane in living cells. Each msfGFP‐fusion protein (0.2 mg mL^−1^) was encapsulated in cell‐size space and its localization was analyzed by confocal microscopy (**Figure** **2**A). No membrane localization was observed when only msfGFP was encapsulated in the cell‐size space. GapA and TpiA seemed to show weak membrane localization, although it was difficult to judge whether the localization was statistically significant from the line profile of the fluorescence intensities. In contrast, msfGFP‐fused proteins (CK, Pgk, PfkA, and Pgi) showed clear membrane localization. Owing to their cytosolic localization in living cells, these proteins are likely to interact with lipid membranes through physicochemical interactions rather than substrate‐specific binding to lipid membranes or membrane proteins. Therefore, we concluded that these bindings are non‐specific.

To analyze the strength of membrane binding of the proteins from a quantitative aspect, we calculated the ratio of the localization of msfGFP fusion proteins in the membrane and cytosol from confocal microscopy images (Figure 2B). If the localization of proteins on the membranes and in the cytosol is in equilibrium, the ratio reflects the membrane affinity constant. Therefore, this estimated ratio was defined as the membrane preference index (Figure S4, Supporting Information). The 99% confidence interval of the index showed that the membrane localization of CK, Pgk, PfkA, Pgi, and GapA was statistically significant. The membrane preference index of each protein was plotted against its ability to generate Min waves (Figure 2C) and suppress Slim‐MinE (Figure 2D). They showed a strong and significant correlation (*p* < 0.05, test of no correlation, *n* = 6), although we failed to find an obvious relationship between the membrane preference index and surface charges of these proteins (Movie S2, Supporting Information).

These results indicated that competition for nonspecific binding to the membrane is a critical factor for Min wave generation and Slim‐MinE suppression in the cell‐size space. Simultaneously, it was indicated that the nonspecific membrane binding is associated with some properties of the lipid membrane interface. If this is the case, the interaction levels depend on the chemical properties of the lipids used for microdroplet formation. To confirm this, we tested microdroplets covered with POPC (neutral charge lipids) and found that Slim‐MinE was not observed in this case (Figure 2E). Furthermore, all the msfGFP‐fusion proteins tested in this study were localized in the cytosol in the case of POPC microdroplets (Figure 2F). These results indicate that the physicochemical properties of the lipid membrane interface are associated with the nonspecific binding of purified proteins to lipid membranes in the cell‐size space.

### 2.3 Quantitative Analysis of the BSA‐Like Effect in Cell‐Size Space Using PURE System

**Figure 3:**
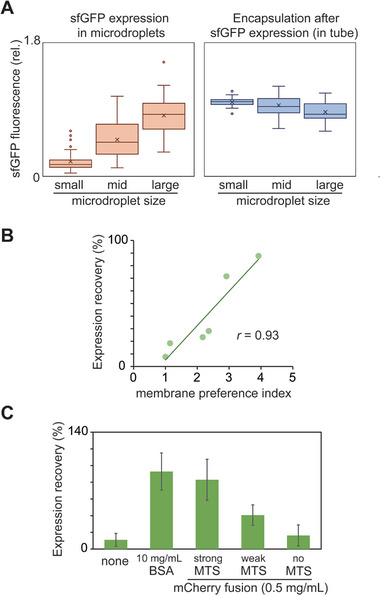
Various purified proteins restore PURE system activity decreased by cell‐size space effect. A) Expression of sfGFP by PURE system in microdroplets of different sizes. Box plots (*n* = 30) show relative fluorescence intensities of sfGFP in microdroplets. Small, mid, and large indicate 20–40 µm, 40–60 µm, and over 60 µm, respectively. (Left) sfGFP levels synthesized in microdroplets. (Right) Encapsulation after sfGFP synthesis in tubes. Expression levels are normalized by the average level of 20–40 µm microdroplets with sfGFP synthesized in a tube. B) Plots of recovery of PURE system activity in microdroplets by supplementation with 1 mg mL^−1^ purified proteins against the membrane preference index of each protein. Recovery levels by each protein are shown (*p* < 0.01, test of no correlation, *n* = 6). C) Recovery of PURE system activity in microdroplets by supplementation with artificial membrane‐binding proteins (0.5 mg mL^−1^ mCherry or its fusion with membrane targeting sequences (*n* = 30, average ± SD). B,C) Recovery levels indicate the ratio of protein expression levels under additional protein conditions in microdroplets to those in tubes.

Our previous study showed that the activity of the transcription‐translation system consisting of purified elements (PURE system) is inhibited in the cell‐size space, and BSA restores the activity similar to the case of Min waves.^[^ ^9b^ ^]^ We investigated the mechanism of the inhibition of PURE system in the cell‐size space and its restoration by BSA. Although the reason PURE system is not active in the cell‐size space covered with *E. coli* polar lipids remained elusive, both transcription and translation were inhibited in the cell‐size space covered with *E. coli* polar lipids (Figure S5, Supporting Information). Because metabolic enzymes should be active irrespective of their localization, a possible explanation is that the membrane localization of some dynamic molecules, such as RNA polymerase and elongation factors, may be inhibited due to membrane localization by the cell‐size space effect. Therefore, as the first step in the investigation of PURE system, we verified whether the interface was associated with the inhibition of PURE system in the cell‐size space. Because the surface‐area‐to‐volume ratio is inversely proportional to the diameter of the sphere cell‐size spaces, the inhibition levels should be lowered in larger microdroplets if the inhibition is derived from interfacial effects. For example, the levels of Slim‐MinE depend on the space size of the microdroplets.^[^ ^6b^ ^]^ To verify this, we analyzed the relationship between the PURE system activity and microdroplet sizes in the absence of additional proteins. The activities of PURE system in small (20–40 µm diameter), medium (40–60 µm diameter), and large (>60 µm diameter) microdroplets were evaluated by expression levels of sfGFP by quantification of fluorescent levels using confocal microscopy (**Figure** **3**A, left panel). Consequently, sfGFP expression was higher in larger microdroplets (*p* < 10^−4^, One‐way ANOVA), showing that the inhibition levels of PURE system in the cell‐size space were lowered by a smaller surface‐area‐to‐volume ratio. To omit the possibility of microscopic artifacts, sfGFP was expressed in tube by PURE system, and the resultant solution was encapsulated in microdroplets to quantify fluorescence levels in microdroplets of the same size ranges by microscopy. In this case, the fluorescence levels in the larger microdroplets were lower than those in the smaller microdroplets (Figure 3A, right panel), although the trend was not statistically significant (*p* > 0.05, one‐way ANOVA). Taken together with our previous study showing that lipid compositions of the cell‐size space affect the activities of PURE system,^[^ ^9b^ ^]^ we concluded that the enhanced interfacial effects in the cell‐size space are associated with the inhibition of PURE system activity.

Next, we tested whether supplementation with purified proteins restored the dysfunction of PURE system in the cell‐size space. Six purified proteins (CK, Pgk, Pgi, PfkA, GapA, and Tpi) at a concentration of 1 mg mL^−1^ were mixed with PURE system, and the restoration levels of PURE system in the cell‐size space were quantified and compared with those of the tube reaction. Similar to the results of the Min wave generation, the restoration levels were significantly correlated with the membrane preference index (*p* < 0.01, no correlation test, *n* = 6)(Figure 3B; Figure S6, Supporting Information). These results also showed that competition for membrane binding among proteins is the key to suppressing the inhibition of PURE system in the cell‐size space.

To further support this conclusion, we used fusion proteins of mCherry with membrane targeting sequences (MTS). Previous studies have shown that membrane preferences differ among MTS species.^[^ ^15^ ^]^ A strong membrane‐binding MTS (2xMreB MTS) or weak MTS (MinD MTS) was fused with mCherry, and the mCherry fusion proteins were added to PURE system and evaluated for their ability to restore the activity of PURE system inhibited by the cell‐size space effect (Figure 3C). Their effects on the restoration of PURE system in the cell‐size space were evaluated using sfGFP expression levels (Figure 3C). mCherry without MTS was used as a control. Consequently, mCherry with 2xMreB MTS and mCherry with MinD MTS strongly and moderately restored the inhibited activity of PURE system in the cell‐size space, respectively. However, mCherry itself did not (Figure 3C). These results demonstrate that the strength of membrane binding, rather than the protein function, is an important factor in determining the strength of the restoration of PURE system activity.

### 2.4 Membrane Competition Model Recapitulates the Regulation of Molecular Localization in Cell‐Size Space

The above results show that various proteins can modulate protein localization, which is critical for the functionality of biochemical systems in the cell‐size space. This ability is related to the level of non‐specific membrane binding of the protein. Notably, increasing the salt concentration (from 150 to 500 mm), which affects Debye‐Hückel screening of the electrostatics (a potential cause of non‐specific interaction with lipid membranes), did not suppress Slim‐MinE, Min wave generation, or the restoration of PURE system activity in the cell‐size space (Figure S7, Supporting Information). Furthermore, the fluidity of the lipids, which affect the asymmetric localization of molecules, was confirmed by fluorescent recovery after photobleaching (Figure S8, Supporting Information). Taken together, we conclude that competition for membrane binding suppresses the localization shift of proteins by the cell‐size space effect, and this suppression is key to ensuring the normal function of biochemical systems in the cell‐size space.

**Figure 4:**
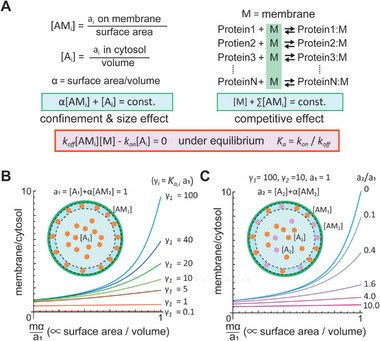
Theoretical model of the cell‐size space effect to explain Slim‐MinE and its suppression by surrounding molecules. A) Framework of the theoretical analysis. Effects by confinement and size are expressed by mass conservation and surface‐area‐to‐volume ratio. Membrane competition and equilibrium are also assumed. B) Theoretical results of the spontaneous lipid membrane localization of a molecule (like Slim‐MinE) in the cell‐size space. C) Theoretical results of localization shift of a Slim molecule by the competition with surrounding molecules in the cell‐size space. B,C) Membrane/cytosol and γ indicate [AM_1_]/[A_1_] and K_a,i_a_1_, respectively (See Experimental Section). *m* is the total site density in the membrane.

This finding suggests that the effects of various proteins on the cell‐size space were caused by competitive interactions between non‐specific binding of proteins to lipid membranes. Here, we explain this phenomenon using a theoretical model that considers simple membrane binding competition with the effects of confinement and cell size. In this model, the membrane is a common and limited resource, and proteins competitively bind to membranes (**Figure** **4**A). The former was expressed by setting the accessible membrane concentration to [M] and the total membrane‐binding sites to *m*. The latter was expressed by assuming that each protein detached from the membrane with an association rate constant (*k*_on_) and a dissociation rate constant (*k*_off_). Confinement was expressed by the mass conservation of molecules, which means that the total amount of each molecule in the cytosol and on the membrane remains constant over time. The size effect was introduced by adding a factor α, corresponding to the surface area to volume ratio, to the mass conservation equation. α is 3/r when considering a spherical space with a radius of r. [A_i_], [AM_i_], and a_i_ indicate the concentration of *i‐th* protein in cytosol, on the membrane, and their total amounts, respectively (Figure 4A). Although Min waves and protein synthesis are non‐equilibrium phenomena, the Slim phenomena of MinE or other GFP fusion proteins can be considered as the result of equilibrium conditions. Under the equilibrium condition, the ratio of [AM_i_] to [A_i_][M] is constant and was expressed as *K*_a_ = *k*_on_/*k*_off_. For the theoretical analysis, all constants and variables were normalized to the total amount of the protein of interest and total membrane‐binding sites (see Experimental Section). By normalization, the constant *K*_a_ multiplied by a_1_ was expressed by γ. It should be noted that although both membrane preference index (Figure 2B) and γ indicate membrane affinities and they show correlation, they do not completely match because membrane preference changes upon *α*, *m*, *a_i_*, [M].

The theoretical model recapitulated the Slim phenomenon that the localization of a single protein encapsulated in cell‐size space shifts to the membrane in the case of γ>1 (Figure 4B). An increase of γ decreases the accessible membrane [M] because more proteins localize on the membrane at higher γ. However, [M] becomes large in smaller spaces, i.e., at larger normalized α (= *m*α/*a_1_*). Therefore, proteins in the cell‐size space prefer membrane localization. A previous study showed that the saturation concentration of MinD on a lipid membrane is ≈10 000 molecules µm^−2^,^[^ ^16^ ^]^ indicating *m* is around this value. Since 1 µm is approximately equal to 600 molecules µm^−3^, the normalized α within cell‐size spaces with a radius of 20, 50, and 100 µm are estimated as ≈5, 2, and 1, respectively. Thus, it is reasonable to assume that the cell‐size space induces the Slim phenomenon in proteins. Non‐dimensionalization of the experimental results of Slim‐MinE obtained in our previous study^[^ ^6b^ ^]^ showed similar profiles of membrane localization of MinE in mα/a_1_ and γ dependent manner (Figure S9, Supporting Information), supporting the notion.

Next, we calculated the localization shift of the protein in the cell‐size space by the supplementation with a surrounding protein. In this case, we set γ_1_ = 100 for the protein of interest (the protein for a_1_), which induces strong membrane localization in cell‐size space, and γ_2_ = 10 for the surrounding protein (the protein for a_2_). Under these conditions, increasing the amount of surrounding protein reduced membrane localization, as observed in the suppression of Slim‐MinE by BSA or the purified proteins (Figure 4C). Theoretical analysis indicates that the relative relationships among four variables (γ_1_, a_1_, γ_2_, a_2_) determine whether the surrounding protein suppresses the membrane localization of the protein of interest or not (Equations S9 and S11, Supporting Information (Notes)). This notion matched the results shown in Figure 1D and explained why the minimum BSA concentration for the activity of PURE system (10 mg mL^−1^) and wave generation by Min system (3 mg mL^−1^) were different. This result shows that the suppression is not due to stronger attachment of the surrounding proteins to the membrane compared to Slim‐MinE. This was also the case when many proteins with much weaker membrane affinities were added; that is, the total concentration of added proteins other than Slim‐MinE was high (see Notes, Supporting Information). These theoretical results show that simple membrane binding competition with the effects of confinement and cell size is sufficient to recapitulate the Slim phenomenon and its suppression.

### 2.5 Additive Effect of Surrounding Proteins on the Lipid Membrane Localization Shift

**Figure 5:**
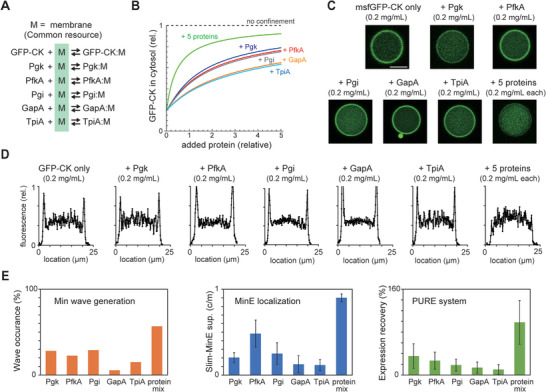
Competition of lipid membrane binding among proteins is a factor to modulate spatiotemporal organization of biomolecules and biochemical activities in cell‐size space. A) Membrane binding competition model. B) Simulation results of localization shifts of GFP‐CK by the addition of each protein or protein mixture. Localization shift of the GFP‐CK in cell‐size space (α = 1, solid lines) or no‐confinement (α = 0, the dashed line) by the addition of individual 5 proteins or all at once at various concentrations were plotted. Relative amounts of GFP‐CK in cytosols are plotted on the y‐axis. The membrane affinity values (γ) were determined by a scaled membrane preference index (Figure 2B) to set the cytosolic fraction of GFP‐CK without surrounding proteins as 0.2. In practice, γ of CK, Pgk, PfKA, Pgi, GapA, and TpiA are 21.0, 14.5, 12.0, 11.0, 5.5, and 5.0, respectively (See Methods, Supporting Information). C) Confocal images of msfGFP‐CK in microdroplets encapsulating purified proteins (0.2 mg mL^−1^ each) and msfGFP‐CK. +5 proteins indicate that Pgk, PfkA, Pgi, GapA, and TpiA were co‐supplemented with msfGFP‐CK. Scale bar indicates 10 µm and all images are shown in the same pixels. D) Line plots of fluorescence intensities in (C). Intensities were normalized by the total fluorescence area of each line profile. E) Effect of the addition of each protein or the mixture of 5 proteins on Min wave occurrence, Slim‐MinE suppression activity, and recovery of sfGFP expression by PURE system in microdroplets. (Left) Effects on Min wave occurrence. Purified proteins or their mixture (0.5 mg mL^−1^) were encapsulated with 1 µm His‐msfGFP‐MinD and 1 µm MinE‐mCherry‐His, and percentages of artificial cells with Min waves are shown (*n* = 104–111). (Middle) Effects on Slim‐MinE suppression activity (c/m). MinE‐mCherry‐His (1 µm) was encapsulated in microdroplets with 0.2 mg mL^−1^ of each purified protein or their mixture (*n* = 10, average ± SD). (Right) Recovery of sfGFP expression by PURE system in microdroplets by 0.2 mg mL^−1^ of each purified protein or their mixture is plotted (*n* = 30, average ± SD). In all panels, the values of the protein mixture are significantly higher than those of other single proteins (*p* < 10^−4^, binominal test for A, and *p* < 10^−6^, unpaired *t*‐test for B and C).

We applied this model to simulate the suppression levels of Slim‐GFP‐CK by surrounding proteins and their mixtures (**Figure** **5**A). The simulation indicated that the suppression level increased in an additive manner with the concentrations of the surrounding proteins, and even at the concentration at which each protein exhibited weak Slim suppression, mixing them had the ability to exhibit high Slim suppression levels (Figure 5B). We verified these theoretical results through microdroplet localization assays. As a result, the low concentration (0.2 mg mL^−1^) of the co‐supplemented proteins (Pgk, PfkA, Pgi, GapA, and TpiA) did not shift the membrane localization of msfGFP‐CK: however, their mixture showed the additive inhibitory effect on membrane localization (Figure 5C,D). This result indicates that spontaneous membrane binding via nonspecific interactions in the cell‐size space was suppressed in an additive manner in a multimolecular environment, as expected in the model proposed above. Furthermore, the effects of adding a mixture of the five surrounding proteins on the generation of Min waves, suppression of Slim‐MinE, and restoration of PURE system activity were analyzed and compared with those of the addition of a single protein. Consequently, an additive effect of the protein mixture was observed in all the cases (Figure 5E). These results support the binding competition model and imply that proteins localized in the membrane can be shifted by changes in the physicochemical properties of the multimolecular environment, such as membrane‐binding strength or protein concentration.

### 2.6 Multimolecular Competition Sets the Threshold of Membrane Localization of Proteins in Cell‐Size Space

Finally, using the binding competition model, we calculated the effect of competition between multiple proteins on protein localization in the cell‐size space. The cytoplasm of living cells is filled with various proteins at concentrations > 100 mg mL^−1^.^[^ ^17^ ^]^ As shown by the assays using fractionated and purified proteins (Figure 1), it was expected that half of the proteins in the cytosol would have the potential to interact with lipid membranes. These proteins prefer membrane localization because of the cell‐size space effect (Figure 4). Our results also indicated that the suppression levels were determined, irrespective of the protein species in an additive manner (Figure 5). This indicates that multimolecular competition suppresses irregular protein localization, which is enhanced by the cell‐size space effect.

**Figure 6:**
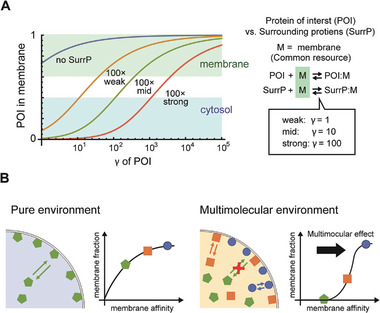
Multimolecular competition modulates protein localization in cell‐size space. A) Simulation results of multimolecular competition effects on protein localization. Localization of the protein of interest (POI) mixed with surrounding proteins (SurrP) exhibiting different membrane affinities (γ = 1, 10, and 100) at 100‐fold higher concentration of the target protein in the cell‐size space (α = 1) was calculated using the membrane competition model. Membrane and cytosolic localization are defined by the localization of ≥60% of the target protein. B) Membrane localization of purified elements alone or with surrounding proteins in the cell‐size space. Proteins with low membrane affinity in purified environments localize in membranes due to the cell‐size space effect. The membrane localization threshold is modulated by multimolecular competition with surrounding proteins. Restricted proteins with high affinity to the membrane can exhibit membrane localization under multimolecular competition such as those found in living cells.

To investigate this, we simulated protein localization in a cell‐size space with surrounding proteins using the binding competition model. In this simulation, we assumed the protein of interest is surrounded by a 100‐fold concentration of proteins with various membrane affinity (γ = 1, 10, and 100). The simulation showed that the threshold membrane affinity of the protein of interest for membrane localization depended on that of the surrounding proteins (**Figure** **6**A). In other words, the membrane localization of the protein of interest is determined by the relative relationship of competition of membrane binding between the protein of interest and the surrounding molecules. These results indicate that multimolecular competition modulates protein localization in cell‐size spaces (Figure 6B). Multimolecular competition works as a modulator of spatiotemporal patterns in cells, such as Min waves, and complex biochemical networks, such as the transcription‐translation system.

## 3 Conclusion

In this study, we investigated why co‐supplementation of proteins is necessary to ensure normal functions of Min system and transcription‐translation system in cell‐size space. We demonstrated that this is based on competitive membrane binding among proteins in cell‐size space (Figures 4 and 5). Furthermore, this competition suppressed the membrane localization enhanced by the cell‐size space effect, and the levels of suppression were in proportion to the strength of the non‐specific membrane binding of the proteins (Figures 3, 4, 5). Using theoretical analysis, we showed that multimolecular competition modulates the localization shift of proteins in the cell‐size space, and this modulation ensures biochemical homeostasis of intracellular spatiotemporal organization and biochemical networks. Because the association constant of BSA with lipid membranes is very weak (≈10^3^ m ^−1^),^[^ ^18^ ^]^ our results indicate that very weak interactions, irrespective of whether they are transient or stable, are sufficient for membrane competition if the concentration is high. These results depicted an unexplored trait of multimolecular environments in cell‐size space: membrane competition of various macromolecules even if they show very weak affinity to lipid membranes in cells sets the threshold of whether proteins can localize on lipid membranes in cells (Figure 6). For example, MinD concentration in living cells is ≈1 µm and is surrounded by >1 mm surrounding proteins which possibly nonspecifically interact with lipid membranes. In this case, MinD should show more than 1000‐times higher membrane affinity than the surrounding proteins show is necessary for the membrane localization. Because surrounding molecules do not affect the localization of MinD at the concentration ranges in this study, it is plausible that MinD shows much stronger membrane affinity than MinE and other surrounding proteins we tested. This multimolecular competition effect emerges from the relative relationship between membrane affinity and concentration of proteins. It appears even at 1–10 mg mL^−1^ of surrounding proteins, and therefore, is different from the macromolecular crowding effect, which only becomes apparent at high concentrations (100–300 mg mL^−1^).

The multimolecular competition effect found in this study was investigated by using the cell‐size space of eukaryotes (10–30 µm), which is larger than *E. coli* cells (1–3 µm). Our theoretical analysis showed that the cell‐size space effect becomes stronger because the surface‐area‐to‐volume ratio is much larger in smaller spaces. Hence, multimolecular effects that suppress membrane localization play an even more important role in smaller spaces. Furthermore, because the membrane affinities of the surrounding proteins depend on the proteome, it is natural that they differ among organelles, cell types, and organisms,^[^ ^19^ ^]^ and even in different cellular conditions. Our findings suggest that the physicochemical environment shaped by multimolecular competition should be considered when understanding the localization of biomolecules in living cells.

Most biochemical studies using purified elements conducted so far have applied only essential elements in test tubes to imitate biological systems.^[^ ^1^ ^]^ However, the results of this study revealed that essential elements alone are not sufficient to reconstitute biochemical systems in cell‐size spaces, and exogenous proteins irrelevant to the system are critical for the assurance of normal molecular organization in such environments. This characteristic is not limited to systems for the spatiotemporal regulation of cell division and transcription‐translation those are demonstrated in this study, because this study showed that the function of multimolecular competition is based on its physical environment rather than the specific functions of proteins. This finding facilitates the reconstitution of biochemical systems within artificial cells and contributes to materials science for manipulating nano/micro spaces because of their similarity to cell‐size spaces.

## 4 Experimental Section

### Plasmid Construction

All plasmids except for the expression of Min proteins were constructed in this study (Table S1, Supporting Information) by iVEC3 method.^[^ ^20^ ^]^ Glycolysis genes (Pgk, FbA, GpmA, Glk, PfkA, Eno, Pgi, PykA, GapA, TpiA) were amplified by PCR using *E. coli* MG1655 genome. CK gene, which is derived from *Gallus gallus*, was amplified from the plasmid used in the original PURE system.^[^ ^12^ ^]^ The amplified genes were cloned into the PCR fragment of pSUMO or pSUMO‐msfGFP by iVEC3 method. For the construction of pSUMO‐MTS_2xmreB_‐mCherry, mCherry gene was cloned into pSUMO vector with MTS_2xmreB_ sequences. MTS_2xmreB_ was designed by following the construct in a previous study ^[^ ^15^ ^]^ and was attached by PCR. For the construction of pET29‐mCherry‐MTS_minD_‐His, MTS_minD_ designed by following the construct in a previous study^[^ ^21^ ^]^ was attached to pET29‐mCherry‐His.^[^ ^22^ ^]^ pET15‐sfGFP‐minD and pET29‐minE‐mCherry‐His were constructed in our previous study.^[^ ^6b^ ^]^ pET29‐LDH‐His for LDH expression was constructed in our previous study^[^ ^23^ ^]^). pET15‐msfGFP‐minD was constructed by V206K mutant of pET15‐msfGFP‐minD.

### Protein Expression and Purification

*E. coli* BL21‐CodonPlus(DE3)‐RIPL cells (Agilent Technologies, Santa Clara, CA, USA) harboring each protein expression plasmid were cultured at 37 °C in an LB medium with 100 µg mL^−1^ ampicillin. Proteins were overexpressed by 1 mm IPTG at OD_600_ = 0.2 and further cultivated at 37 °C for 3 h. Cells overexpressing the proteins were harvested by centrifugation at 4 °C for 2 min at 8000×*g*.

For His‐msfGFP‐MinD purification, cells overexpressing His‐msfGFP‐MinD by pET15‐msfGFP‐minD (V206K mutant of pET15‐msfGFP‐minD) were suspended in LS Buffer (50 mm Tris‐HCl pH 7.6, 300 mm NaCl, 1 mm DTT, 0.1 mm PMSF, 10 mm imidazole, and 0.2 mm ADP) and were disrupted by ultrasonication using SONIFIER 250 (Branson, Danbury, CT, USA) for 30 min (total ON: 10 min) in ice water bath. After centrifugation at 4 °C for 30 min at 20000×*g*, the supernatant was collected and mixed with cOmplete His‐Tag Purification Resin (Roche, Basel, Switzerland). After shaking the mixture at 4 °C for 30 min, the mixture was loaded into Poly‐Prep Chromatography Columns (Bio‐Rad, Hercules, CA, USA) and washed with WS Buffer (50 mm Tris‐HCl pH 7.6, 300 mm NaCl, 0.1 mm PMSF, 10% glycerol, 0.1 mm EDTA, and 20 mm imidazole). His‐msfGFP‐MinD was eluted with EL Buffer (50 mm Tris‐HCl pH 7.6, 300 mm NaCl, 0.1 mm PMSF, 10% glycerol, 0.1 mm EDTA, and 250 mm imidazole). The buffer was replaced with Storage Buffer (50 mm HEPES‐KOH pH 7.6, 150 mm GluK, 10% glycerol, 0.1 mm EDTA, and 0.2 mm ADP) using AmiconUltra‐4 (Merck Millipore, Billerica, MA, USA) and then concentrated to 100 µm using AmiconUltra‐0.5 30 k filters. For purification of MinE‐mCherry‐His, pET29‐minE‐mCherry‐His was used for protein expression and ADP was omitted from the buffers. MinE‐mCherry‐His used in this study is from the same lot as used in our previous study.^[^ ^6b^ ^]^

For purification of other proteins, Ni Sepharose 6 Fast Flow (GH Healthcare, Chicago, IL, USA) was used instead of cOmplete His‐Tag Purification Resin. STI buffer (50 mm Tris‐HCl pH 7.6, 500 mm NaCl, and 40 mm Imidazole) was used instead of LS buffer and WS buffer, and EL2 buffer (50 mm Tris‐HCl pH 7.6, 500 mm NaCl, and 500 mm Imidazole) was used instead of EL buffer. The buffer for proteins purified by Ni sepharose was replaced with Ulp1 processing Buffer (40 mm Tris‐HCl pH7.6, 200 mm NaCl, and 10% Glycerol) using PD‐10 Columns (GE Healthcare), and SUMO‐tag was cut by homemade Ulp1^[^ ^9b^ ^]^ supplemented in the solution. After the Ulp1 treatment, the His‐SUMO fragment and Ulp1 were removed by Ni sepharose, and the proteins in the flowthrough fraction were collected. The buffer of the purified proteins was replaced with RE Buffer (25 mm Tris‐HCl pH7.6, 150 mm GluK, and 5 mm GluMg) using AmiconUltra‐4 filters (Merck Millipore) or further purified by Q Sepharose High Performance (GE Healthcare) or SP Sepharose High Performance (GE Healthcare) for PURE system reactions. For Q Sepharose, proteins were eluted by 20 mm HEPES‐KOH pH 7.6 containing 100–500 mm KCl. For Q Sepharose, proteins were eluted by 20 mm HEPES‐KOH pH 7.6 containing 150–900 mm GluK. The solution was diluted to fivefold with ultrapure water and concentrated with AmiconUltra‐0.5 filters (Merck Millipore).

Protein concentrations were determined by CBB stained gels after SDS‐PAGE and BCA assay using the Pierce BCA Protein Assay Kit (Thermo Fisher Scientific, Waltham, MA, USA), and all samples were stored at −80 °C. Details of the protocol are reported in the literature.^[^ ^24^ ^]^

### Preparation of Microdroplets

Chloroform‐dissolved 25 mg mL^−1^ *E. coli* polar lipid extract (Avanti, Alabaster, AL, USA) and 1‐palmitoyl‐2‐oleoyl‐glycero‐3‐phosphocholine (POPC) (Avanti) stored at −30 °C were heated to room temperature and 20 µL of the solution was transferred to a glass tube. The lipid in chloroform was dried uniformly with argon gas, and mineral oil (Nacalai Tesque) was added to 1 mg mL^−1^ of lipids at the final. The lipid mixture was sonicated for 90 min at 60 °C using Bransonic (Branson) and then stirred for 1 min with vortex. Microdroplets were prepared by adding an inner solution to the lipid mixture at a ratio of 50:1 and by tapping them 10–15 times. Because the distribution of microdroplet sizes was not uniform by the tapping method, microdroplets with a defined range of sizes were randomly selected for the analyses.

### Observation of Min Wave Generation and Min Protein Localization

Mixtures of 1 µm MinD (His‐msfGFP‐MinD), 1 µm MinE (MinE‐mCherry‐His), 2.5 mm ATP, and 0.2–10 mg mL^−1^ proteins (purified proteins or cell extract with or without fractionation) in RE buffer were prepared as the inner solution, and microdroplets were prepared as described above. The final GluK concentration was raised to 500 mm if indicated. Because cell extract shows strong ATPase activity, ATP regeneration system (0.4 mg mL^−1^ creatine kinase and 80 mm creatine phosphate) was added to the fractions including the positive control (cell extract without fractionation) and the negative control (no cell extract). 20 µL of microdroplet solution was placed in two glass coverslip slits with a double‐sided tape as spacers,^[^ ^24^ ^]^ and Min waves were observed in microdroplets using a fluorescence microscope (Axiovert Observer Z1, Carl Zeiss, Jena, Germany). For the analysis, microdroplets with a diameter of 10–30 µm were randomly picked up. Time‐lapse images were taken at 10 s intervals for 5 min, and Min wave generation was assessed by the localization of MinD. It should be noted that MinE was supplied in the reaction mixture but its localization was not used for the assessment of Min wave generation. Microdroplets with 45° or more movement of MinD within 5 min were determined as the microdroplets with Min waves. Fiji software (National Institutes of Health, Bethesda, MD, USA) was used to analyze the obtained images.

### Analysis of Slim‐MinE Suppression Activity and Membrane Preference Index

Microdroplets were prepared in the same manner as described above. To observe the localization shifts of Min proteins, a mixture containing 1 µm MinD (His‐msfGFP‐MinD) or 1 µm MinE (MinE‐mCherry‐His) and 0.2–10 mg mL^−1^ purified proteins in RE buffer was prepared as the inner solution. The final GluK concentration was raised to 500 mm if indicated. For MinD localization assay, 2.5 mm ATP was added to the mixture. The microdroplet solution was placed in a slit between slide glasses and the localization of the proteins was observed with a confocal laser‐scanning microscope. Localization of MinD or MinE in microdroplets with a diameter of 10–30 µm was analyzed by using a confocal laser‐scanning microscope FV1000 (Olympus, Tokyo, Japan). Line plots of each microdroplet were obtained using Fiji software and the cytosolic/membrane localization ratio (c/m ratio) was calculated as follows. Membrane intensities (m) were obtained as the highest intensity of two membrane edge peaks, and cytosol intensities (c) were obtained as the average intensities of 10 pixels around the center position (Figure S3, Supporting Information). To reduce noise signal, line plots were smoothed by simple intensity averages.

For other proteins, 0.2–1 mg mL^−1^ of msfGFP fused proteins in RE buffer were used as the inner solution. Microdroplets with a diameter of 10–30 µm were analyzed by using the confocal laser‐scanning microscope. Similarly to the c/m ratio analysis, intensities of membranes and cytosols were quantified from line plots smoothed by simple intensity average, and the obtained ratio (m/c) was calculated as the membrane preference index (Figure S4, Supporting Information).

### Protein Expression Using PURE System Within Microdroplets

Among commercial PURE systems, PUREfrex ver.1.0 (Gene Frontier, Chiba, Japan) was used in this study. As the template DNA for sfGFP expression, 1 nm PCR product of DNA encoding sfGFP with T7 promoter (amplified from pET29‐sfGFP^[^ ^25^ ^]^) was used. The template DNA and 0.2–10 mg mL^−1^ purified proteins were added to PURE system in test tubes on ice. The final GluK concentration was raised to 500 mm if indicated. For protein expression within microdroplets, microdroplets were prepared by using a portion of the reaction mixture as the inner solution and the microdroplet solution was dropped onto a glass‐based dish (AGC TECHNO GLASS, Shizuoka, Japan). For protein expression, the microdroplets were incubated for 4 h at 37 °C. In the case of the tube reactions, microdroplets were prepared by using the resultant mixture after 4 h in tube reaction at 37 °C as the inner solution. Fluorescent images were obtained by using a fluorescence microscope (Zeiss Axio observer Z1), and the amount of protein synthesis was estimated by measuring the fluorescence intensity in microdroplets using Fiji software. If not noted, microdroplets with a diameter of ≈15 µm were analyzed. Fluorescent images were acquired using the confocal microscope in the case of quantification of the cell‐size dependence of PURE system activity.

### Theoretical Analysis of Binding Competition Model

[*M*], [*A_i_*], and [*AM_i_*] are the density of free sites on the membrane, concentration of the protein indexed by i in bulk, and concentration of the protein indexed by i [index of each type of protein (*i* = 1 for the target protein, *i*⩾2 for other proteins) on the membrane], respectively. *a_i_*_,_ *m*, *k*_*on*,*i*_, and *k*_*off*,*i*_ are the total concentration of the protein indexed by *i*, total site density on the membrane, on‐rate of the protein indexed by i onto the membrane, and off‐rate of the protein indexed by *i* from the membrane. α is surface‐area‐to‐volume ratio, which is 3/*r* in the case of inside spherical spaces like microdroplets used in this study. Kinetic equilibrium, mass conservation for each I, and total membrane sites were defined as

(1) $$-k_{on,i}\;\left[A_{i}\right]\left[M\right]+k_{off,i}\;\left[AM_{i}\right]=\;0$$

(2) $$a_{i}=\alpha \left[AM_{i}\right]+\left[A_{i}\right]$$

(3) $$m=\sum_{i}\left[AM_{i}\right]+\left[M\right]$$

respectively. The variables and parameters were normalized as follows: $\frac{\left[A_{i}\right]}{a_{1}}\to \left[A_{i}\right]$, $\frac{\left[M\right]}{m}\to \left[M\right]$, $\frac{\left[AM_{i}\right]}{m}\to \left[AM_{i}\right]$, $\frac{m\alpha }{a_{1}}\to \alpha$, $\frac{k_{on,i}}{k_{off,i}}a_{1}\to \gamma_{i}$

By the normalization, Equations ((1), (2), (3)) becomes,

(4) $$-\gamma_{i}\;\left[A_{i}\right]\left[M\right]+\;\left[AM_{i}\right]=\;0$$

(5) $$1\;=\;\alpha \left[AM_{1}\right]+\left[A_{1}\right],\;\;\;a_{i}=\;\alpha \left[AM_{i}\right]+\left[A_{i}\right]\left(i\geq 2\right)$$

(6) $$1\;=\sum_{i}\left[AM_{i}\right]\;+\;\;\left[M\right]$$

The equations were solved by Mathematica (Wolfram Research, Inc., Mathematica, Version 13.2, Champaign, IL). More details are described in Notes (Supporting Information).

### Other Methods

The methods for the preparation of BSA, cell extract preparation and its fractionation, surface charge calculation, PURE system analysis, and FRAP analysis are described in Methods (Supporting Information).

### Statistical Analysis

Normalization method of data, data presentation such as mean ± SD), sample sizes (*n*) for each statistical analysis, and statistical methods used to assess significant differences with sufficient details are described in the manuscript or the legends of the corresponding Figures. Software used for statistical analysis are Microsoft Excel and R‐4.3.2 for Windows.

## Conflict of Interest

The authors declare no conflict of interest.

## Supporting information

Supporting Information

Supplemental Movie 1

Supplemental Movie 2

## Data Availability

The data that support the findings of this study are available from the corresponding author upon reasonable request.
