# Supplementary material for: Multimolecular Competition Effect as a Modulator of Protein Localization and Biochemical Networks in Cell‐Size Space
Source: Adv Sci (Weinh). 2023 Dec 6;11(6):2308030. doi: 10.1002/advs.202308030 (PMC10853730; doi:10.1002/advs.202308030)
Supplement: Supplementary file 1 — Supporting Information [file ADVS-11-2308030-s002.pdf]

ADVANCED SCIENCE
Open Access

Supporting Information

for *Adv. Sci.*, DOI 10.1002/advs.202308030

Multimolecular Competition Effect as a Modulator of Protein Localization and Biochemical Networks in Cell-Size Space

*Saki Nishikawa, Gaku Sato, Sakura Takada, Shunshi Kohyama, Gen Honda, Miho Yanagisawa, Yutaka Hori, Nobuhide Doi, Natsuhiko Yoshinaga and Kei Fujiwara**

Supporting Information

Multimolecular Competition Effect as a Modulator of Protein Localization and Biochemical Networks in Cell-size Space

*S. Nishikawa, G. Sato[†], S. Takada[†], S. Kohyama[†], G. Honda, M. Yanagisawa, Y. Hori, N. Doi, N. Yoshinaga, K. Fujiwara**

[†] These authors are contributed equally to this work (co-2nd authors).

* Corresponding author. Kei Fujiwara (fujiwara@bio.keio.ac.jp)

Contents:

Supplementary Notes

Supplementary Methods

Supplementary Table S1. Plasmids constructed in this study for protein expression

Supplementary Figure S1. Illustration of cell-size space used in this study.

Supplementary Figure S2. Localization shift of MinD by the addition of 10 mg/mL purified proteins.

Supplementary Figure S3. Localization of GFP-fusion proteins by leaky expression in living cells.

Supplementary Figure S4. Definition of Slim-MinE suppression (c/m) and membrane preference index.

Supplementary Figure S5. Inhibition of transcription and translation in cell-size space covered with *E. coli* polar lipids.

Supplementary Figure S6. Recovery of PURE system activity in microdroplets by supplementation with 1 mg/mL purified proteins.

Supplementary Figure S7. Effect of high salt concentration on Slim-MinE, Min wave generation, and protein expression by PURE system.

Supplementary Figure S8. Fluidity of polar lipids covering artificial cells.

Supplementary Figure S9. Experimental results of Slim-MinE dependence on cell-size space.

Supplementary Movie S1. Min waves reconstituted in artificial cells (100 mg/mL BSA)

Supplementary Movie S2. Surface charges of proteins that show non-specific binding to the lipid membranes.

Supplementary Notes

Theoretical analysis for the membrane competition model in the cell-sized space

$[M]$, $[A_i]$, and $[AM_i]$ are density of free sites on membrane, concentration of the protein indexed by *i* in cytosol, and concentration of the protein indexed by *i* [index of each type of proteins (*i*=1 for the target protein, *i*⩾2 for other proteins) on the membrane], respectively. a_i, m, $k_{on,i}$, and $k_{off,i}$ are total concentration of the protein indexed by *i*, total site density on the membrane, on-rate of the protein indexed by i onto the membrane, off-rate of the protein indexed by *i* from the membrane. $\gamma_i = \frac{k_{on,i}}{k_{off,i}}$ and α = surface area/volume. We should note that $\alpha \propto 1/r$ in the case of interior of spherical spaces, such as the artificial cells used in this study.

Kinetic equilibrium, mass conservation for each *i*, and total membrane sites were defined as

$$-k_{on,i}\,[A_i][M] + k_{off,i}[AM_i] = 0 \qquad (1)$$

$$a_i = \alpha[AM_i] + [A_i] \qquad (2)$$

$$m = \sum_i [AM_i] + [M] \qquad (3)$$

, respectively.

The variables and parameters were normalized as follows :

$$\frac{[A_i]}{a_1} \rightarrow [A_i]$$

$$\frac{[M]}{m} \rightarrow [M]$$

$$\frac{[AM_i]}{m} \rightarrow [AM_i]$$

$$\frac{m\alpha}{a_1} \rightarrow \alpha$$

$$\frac{k_{on,i}}{k_{off,i}} a_1 = K_{a,i} a_1 \rightarrow \gamma_i$$

By the normalization, equations (1)-(3) becomes,

$$-\gamma_i\,[A_i][M] + [AM_i] = 0 \qquad (4)$$

$$1 = \alpha[AM_1] + [A_1],\ a_i = \alpha[AM_i] + [A_i](i \geq 2) \qquad (5)$$

$$1 = \sum_i [AM_i] + [M] \qquad (6)$$

From (4)-(6),

$$[AM_i] = \frac{\gamma_i a_i [M]}{\alpha \gamma_i [M] + 1} \quad (7)$$

$$[A_i] = \frac{a_i}{\alpha \gamma_i [M] + 1} \quad (8)$$

, and $[M]$ is the solution of

$$1 = \sum_i \frac{\gamma_i a_i [M]}{\alpha \gamma_i [M] + 1} + [M] \quad (9)$$

Cytosol fraction and membrane/cytosol are defined as

$$\text{Cytosol fraction} := \frac{[A_1]}{a_1} = [A_1] \quad (10)$$

$$\frac{membrane}{cytosol} := \frac{[AM_1]}{[A_1]} = \gamma_1 [M] \quad (11)$$

From the equations (9), we can calculate $[M]$. When there is only one protein (for example, only MinE with $i = 1$), we may have an explicit solution of $[M]$. For more than or equal to two proteins, we solve equation (9) by using Mathematica (Wolfram Research, Inc., Mathematica, Version 13.2, Champaign, IL). In the analysis throughout this study, the solution satisfying $[M] > 0$ was selected.

In the case of $\alpha = 1$, membrane fraction (%) of the protein indexed by i is $[AM_i]$, because $[AM_i] + [A_i] = 1$. Therefore, we plotted $[AM_1]$ as the membrane fraction (%) in Fig. 6A.

For the protein crowder simulation in Fig. 5B, γ_i were determined by the membrane preference index (Fig. 2B). In practice, γ_i were $\gamma_{CK} = 4.2s$ for CK, $\gamma_{Pgk} = 2.9s$ for Pgk, $\gamma_{PfkA} = 2.4s$ for PfkA, $\gamma_{Pgi} = 2.2s$ for Pgi, $\gamma_{GapA} = 1.1s$ GapA, and $\gamma_{TpiA} = 1.0s$ for TpiA. These γ_i were determined by the membrane preference index (Fig. 2B). Since the membrane preference index does not match the absolute membrane affinity constant, the γ_i was multiplied by a hyperparameter s. In this study, we set s=5 to set cytosolic fraction of GFP-CK without crowders as 0.2. For the calculation, the equation (9) was solved by using $a_1 = 1$, and a_i $(i \geq 2)$ was varied (x-axis in Fig. 5B). In the case of the single crowder, γ_2 was the corresponding γ of the crowder (For example, $\gamma_2 = \gamma_{Pgk}$ in the case of Pgk addition). In the case of the 5 protein crowder, γ_{Pgk}, γ_{PfkA}, γ_{Pgi}, γ_{GapA}, γ_{TpiA} were set as γ_2, γ_3, γ_4, γ_5, γ_6, and a_i $(i \geq 2)$ was the same irrespective of i and was varied (x-axis in Fig. 5B).

The case of the protein of interest surrounded by a large number of proteins with weak affinity to lipid membrane

As a limiting case, when other proteins bind weakly to lipid membranes, namely $\gamma_i \ll 1$ (to be precise, $\alpha\gamma_i[M] \ll 1$), the equation (9) becomes

$$1 = \frac{\gamma_1[Mw]}{\alpha\gamma_1[Mw]+1} + (1 + \textstyle\sum_{i=2}\gamma_i a_i)\ [Mw] \qquad (9a)$$

. In this case, the protein of interest ($i = 1$) detaches from the membrane if the sum of all other proteins makes enough contribution $\sum_{i=2}\gamma_i a_i \gtrsim 1$ (compare to the case only with $i = 1$). This gives a useful intuition of why other proteins can compete with the protein of interest when the number of different proteins is large and the concentration of each protein is large enough.

Supplementary Methods

Preparation of BSA

For preparation of BSA (A6003, Sigma-Aldrich, St. Louis, MO, USA), powders of the proteins was dissolved in MilliQ water and then the buffer was replaced with RE buffer (for Min wave analysis and protein localization assays) or ultrapure water (for PURE system assays) using AmiconUltra-0.5 filters.

Cell extract preparation and its fractionation

E. coli BL21-CodonPlus(DE3)-RIPL was inoculated into 3 mL of LB medium (without antibiotics) and pre-cultured at 37°C overnight. The pre-cultured medium was added to 1% of 250 mL LB medium (without antibiotics) and was cultivated at 37°C with 180 rpm shaking to reach OD600=0.7. The cells were collected by 8,000×g centrifugation for 2 min at 4°C. The cell pellet was suspended in 4 mL of Lysis buffer (25 mM Tris-HCl pH 7.6, 250 mM NaCl, and 10 mM GluMg) to 1 g of precipitated cells. The cells were disrupted by sonication using Sonifier 250 at output 3, duty 30% for 30 min. The supernatant of the solution after sonication by 30,000×g centrifugation at 4°C for 30 min was collected as the soluble fraction. The soluble fraction incubated at 37°C for 30 min to remove genomic DNA and RNA was then centrifuged at 4°C for 30,000 x g for 30 min. The supernatant after the centrifugation was obtained as the cell extracts and was concentrated and exchanged into Reaction buffer that is used for protein purification. The cell extracts were stored at -80°C. The protein concentration in the cell extract was quantified using the Pierce BCA Protein Assay kit (Thermo Fisher Scientific).

Cell extract were fractionated by anion exchange using Q-sepharose or gel filtration using Sephacryl HR S-300. For anion exchange, cell extract was diluted with 5-fold volume of 50 mM Tris-HCl

(pH7.6), and then, the solution was fractionated by HiTrapQ HP using buffer gradient of 50 mM Tris-HCl with 50 to 1050 mM NaCl (20 fractions). For gel filtration, 20 μL of 50 mg/mL cell extract was applied to 2 mL Sephacryl HR S-300 equilibrated with RB buffer [50 mM Tris-HCl (pH7.6), 250 mM NaCl]. Fractions were obtained by addition of 2 mL RB buffer (20 fractions). The buffers for the fractionated cell extract were exchanged with RE buffer using Amicon Filter 3k.

Surface charge calculation

Surface charge of the proteins were calculated by PyMOL(The PyMOL Molecular Graphics System, Version 2.0 Schrödinger, LLC) and its APBS (the Adaptive Poisson-Boltzmann Solver) plugin. PDB ID are as follows: CK(2crk), Pgk(1zmr), Pgi(3nbu), PfkA(1pfk), GapA(1dc3), and TpiA(4iot).

Activity analysis of transcription and translation of PURE system in the microdroplet

To evaluate the transcription levels, a molecular beacon that bind transcribed RNA was used. In this experiments, mCherry gene under T7 promoter was transcribed in the microdroplet covered with *E. coli* polar lipids or POPC. The sequence of molecular beacon we used is (FAM-TGCTGCACCCTTCAGGGAAGCTCAGGCAGCA-BHQ1).

To investigate the translation levels, mRNA of sfGFP was used as the template for PURE system reaction. The mRNA of sfGFP was prepared by T7 RiboMAX™ Express Large Scale RNA Production System (Promega) and purified by RNeasy (Qiagen). It should be noted that T7RNAP was not omitted from PURE system. The sfGFP DNA in PURE system reactions (see Methods) were replaced with 1 μM mRNA of sfGFP, and was transcribed in the microdroplet covered with *E. coli* polar lipids or POPC. Levels of transcription and translation were evaluated by using the fluorescence microscope and ImageJ as the same as sfGFP expression experiments (see Methods).

FRAP analysis

One μL of 0.1 mg/mL Rhodamine-DMPE (Avanti) in chloroform was mixed with 40 μL of 25 mg/mL *E. coli* polar lipids in chloroform. The lipid in chloroform was added to 1 mL mineral oil (Nacalai Tesque), and was sonicated at 60°C for 90 min. The obtained lipid solution was used for the microdroplet formation described in Methods. As the inner solution for the microdroplet, 100 mg/mL BSA in RE buffer was used. A confocal laser-scanning microscope was used (FV1200; Olympus) for fluorescence recovery after photo-bleaching (FRAP) analysis. The fluorescence recovery of Rhodamine-DMPE with *E. coli* polar lipids in the membranes were measured by the standard protocol of FV1200 using tornado bleaching of a circular area as shown Supplementary Fig. S7.

Supplementary Table S1. Plasmids constructed in this study for protein expression

Gene	Plasmid	Plasmid (fused with GFP)
Glucokinase (Glk)	pSUMO-BH-glk	-
Phosphoglucose isomerase (Pgi)	pSUMO-BH-pgi	pSUMO-msfGFP-pgi
6-phoshofructokinase 1 (PfkA)	pSUMO-BH-pfkA	pSUMO-msfGFP-pfkA
Fructose bisphosphate aldolase class 2 (FbaA)	pSUMO-BH-fbaA	-
Triosephosphate isomerase (TpiA)	pSUMO-BH-tpiA	pSUMO-msfGFP-tpiA
Glyceraldehyde 3-phosohate dehydrogenase (GapA)	pSUMO-BH-gapA	pSUMO-msfGFP-gapA
Phosphoglycerate kinase (Pgk)	pSUMO-BH-pgk	pSUMO-msfGFP-pgk
2,3-bisphoglycerate-dependent phosphoglycerate mutase (GpmA)	pSUMO-BH-gpmA	-
Enolase (Eno)	pSUMO-BH-eno	-
Pyruvate kinase 2 (PykA)	pSUMO-BH-pykA	-
Creatine kinase (CK)	-	pSUMO-msfGFP-CK
MTS_{2xmreB}-mCherry-	pSUMO-MTS_{2xmreB} -mCherry	
mCherry-MTS_{minD}	pET29-mCherry-MTS_{minD}-His	

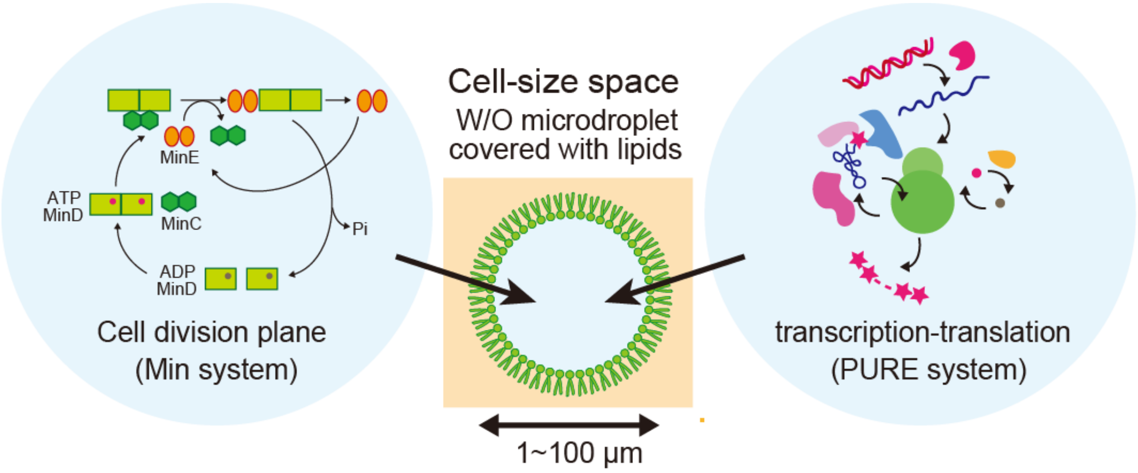

Supplementary Figure S1. Schematic representation of cell-size space used in this study. Water-in-oil (W/O) microdroplets covered with lipids were used for investigating the effect of cell-size space (1-100 μm) on biochemical systems. Throughout this study, *E. coli* polar lipids were used for cell-size space formation except in the case specifically noted. As the biochemical systems, Min system and PURE system were used. It should be noted that the reaction network of Min system is illustrated based on a realistic model but the cartoon of PURE system is an abstract image.

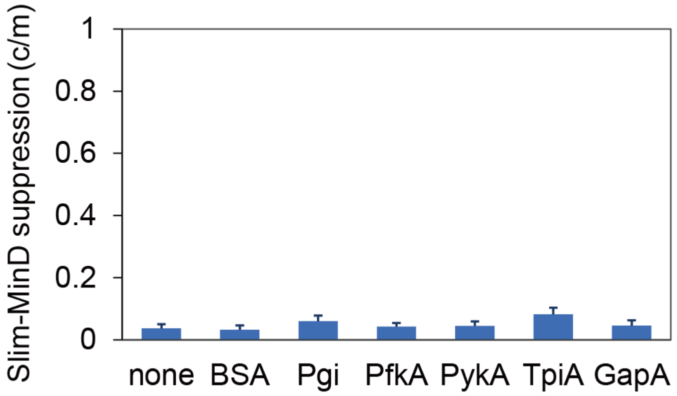

Supplementary Figure S2. Localization shift of MinD by the addition of 10 mg/mL purified proteins.

Localization of MinD in microdroplets encapsulating 1 μM His-msfGFP-MinD (Right) with 10 mg/mL proteins indicated were shown by c/m of MinD (n = 10, average ± SD). We should note that MinE is absent in this experiment.

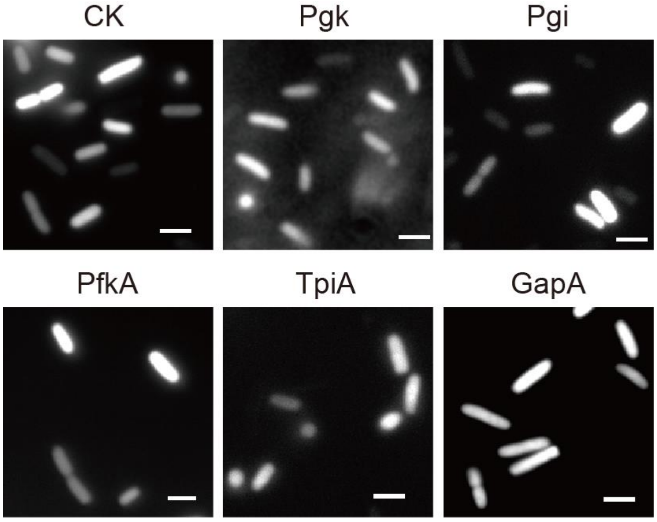

Supplementary Figure S3. Localization of GFP-fusion proteins by leaky expression in living cells. Fluorescence images of living cells harboring the plasmid for each GFP-fusion protein were shown. Scale bars indicate 20 μm.

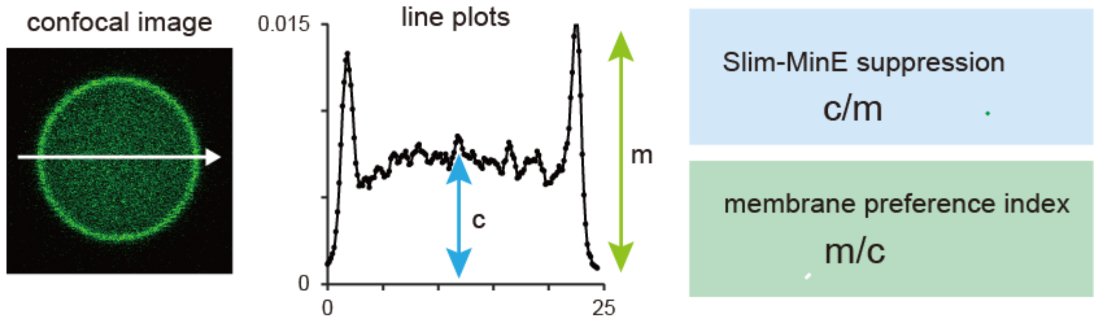

Supplementary Figure S4. Definition of Slim-MinE suppression (c/m) and membrane preference index.

Fluorescent intensities obtained by confocal microscopy are converted to c/m or m/c. The c/m ratio is the index to indicate the suppression levels of spontaneous MinE localization (Slim-MinE) as described in our previous studies (S Kohyama et al., eLife 2019, nanoscale 2020). We should note the "c" and "m" are averages of the fluorescent intensities around the center of the artificial cells and that at the two edges. In the case of no obvious edge, maximum intensities around the edge were used to estimate "m". It should be noted that, although the individual values of m/c and 1/(c/m) are the same, their averages and standard deviations are different.

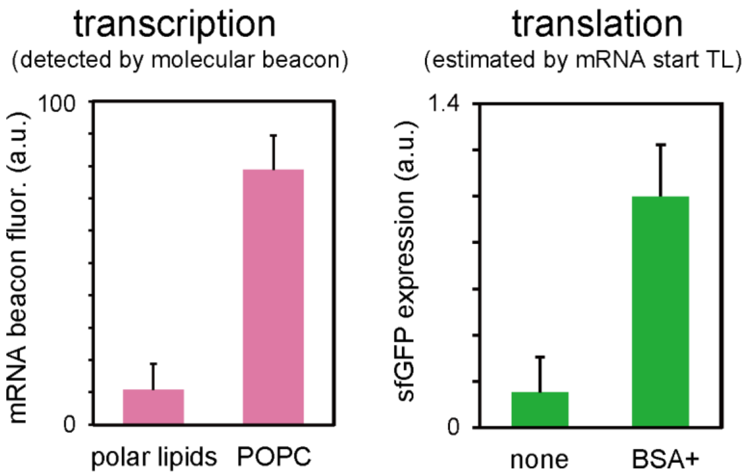

Supplementary Figure S5. Inhibition of transcription and translation in cell-size space covered with *E. coli* polar lipids.

Transcription was evaluated by the fluorescence intensities of a DNA beacon that binds mCherry mRNA transcribed by T7 RNA polymerase in PURE system. Polar lipids and POPC indicate the lipid covering artificial cells. Means and SD are shown (n= 30). The difference in average is significant (unpaired t-test, $p<10^{-10}$). Translation is evaluated by sfGFP expression of PURE system using sfGFP mRNA as the template. None and BSA+ indicate normal PURE system without or with 10 mg/mL BSA. Means and SD are shown (n= 25 for none, and n=42 for BSA+). The difference in average is significant (unpaired t-test, $p<10^{-10}$).

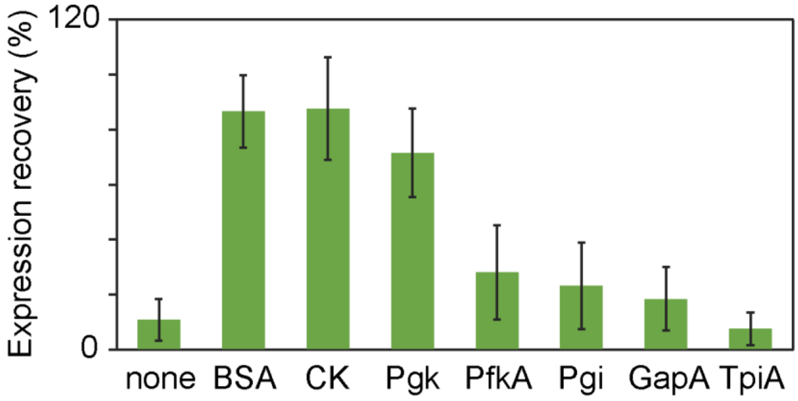

Supplementary Figure S6. Recovery of PURE system activity in microdroplets by supplementation with 1 mg/mL purified proteins.

Recovery of PURE system activity in microdroplets by supplementation with 1 mg/mL purified proteins. Recovery levels indicate the ratio of protein expression levels under additional protein conditions in microdroplets to those in tubes (n=30, average ± SD).

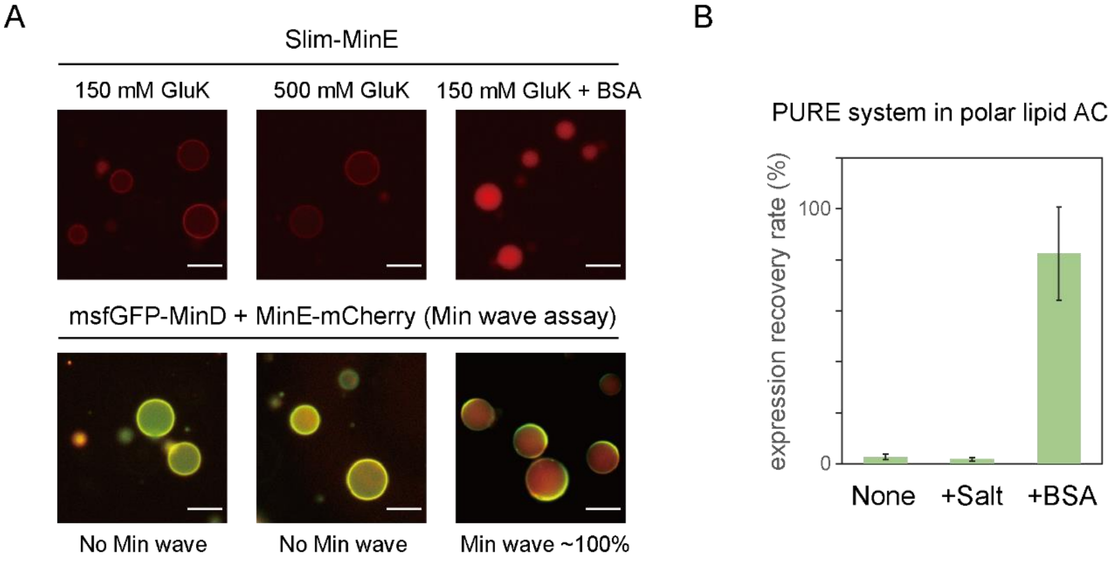

Supplementary Figure S7. Effect of high salt concentration on Slim-MinE, Min wave generation, and protein expression by PURE system.

(A) Slim-MinE and Min wave generation under low (150 mM GluK) and high salt conditions (500 mM GluK) are shown. Slim-MinE was tested by encapsulating 1 µM MinE-mCherry in artificial cells covered with polar lipids. Min wave generation was tested by encapsulating 1 µM msfGFP-MinD and 1 µM MinE-mCherry in artificial cells covered with polar lipids. No Min wave indicates that Min waves are observed in less than 1% of artificial cells. For these tests, GluK concentration in RE buffer (Methods) was varied (150 mM or 500 mM). Scale bars indicate 20 µm. (B) Expression recovery rates of sfGFP expression by PURE system under the high salt condition. Means and SD are shown (n=30). "+Salt" and "+BSA" indicate that 500 mM GluK or 10 mg/mL BSA was added to the PURE system. The difference between "+Salt" and "+BSA" is significant (unpaired t-test, $p<10^{-10}$). The difference between "None" and "+Salt" is also significant (unpaired t-test, $p<10^{-3}$), although the recovery rate of "+Salt" is lower than that of "None".

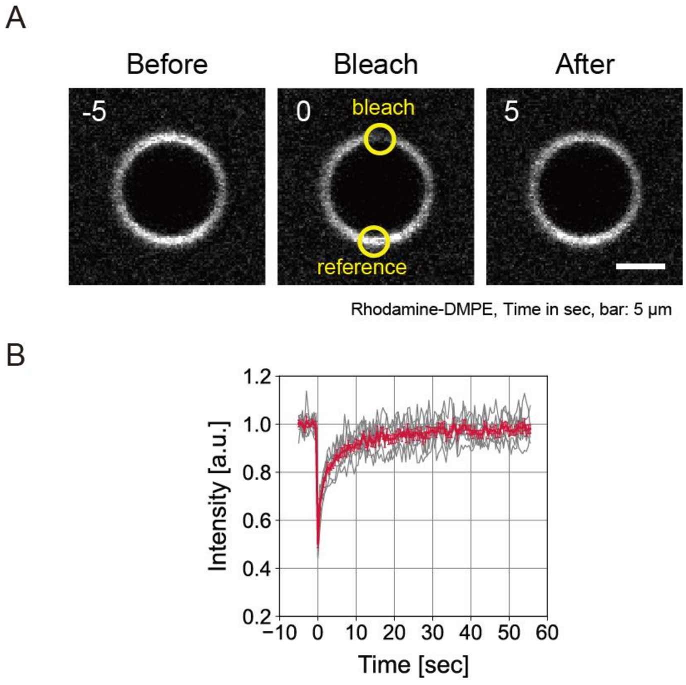

Supplementary Figure S8. Fluidity of polar lipids covering artificial cells.
(A) Fluorescence images of lipids covering a microdroplet before and after photo-bleaching. *E. coli* polar lipids were mixed with Rhodamine-DMPE for the evaluation of fluidity. (B) Intensity plots of fluorescence after photobleaching (FRAP) of lipids covering an artificial cell. Intensities were normalized by those at the reference spot shown in (A). Gray lines indicate the plots for individual artificial cells, and the red line indicates their average (n=7).

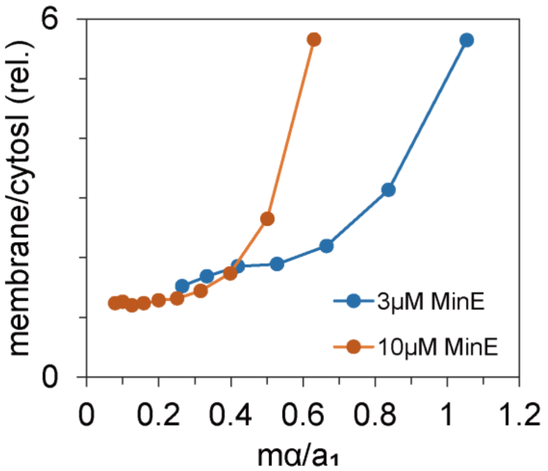

Supplementary Figure S9. Experimental results of Slim-MinE dependence on cell-size space. X-axis (membrane/cytosol) is an inverse of c/m of MinE obtained in a previous study (1). Y-axis is the estimated value of $m\alpha/a_1$ in Figure 4B. For the $m\alpha/a_1$ estimation, *m* and a_1 are set to 10,000 molecules/μm^2 and MinE concentration (molecules/μm^3), respectively. Because $\gamma=K_a a_1$, γ at 10 μM MinE is ~3.33-times higher than γ at 3 μM MinE.

Supplementary References

1. S. Kohyama, N. Yoshinaga, M. Yanagisawa, K. Fujiwara, N. Doi, Cell-sized confinement controls generation and stability of a protein wave for spatiotemporal regulation in cells. eLife 8, e44591-e44591 (2019).

Supplementary Movie S1. Min waves reconstituted in artificial cells (100 mg/mL BSA). The reaction condition was 1μM msfGFP-MinD (Green) and 1μM MinE-mCherry (Red) supplemented with 100 mg/mL BSA.

Supplementary Movie S2. Surface charges of proteins that show non-specific binding to the lipid membranes. Electric charges are shown by color bar. Details are described in Methods.
